# Efficacy of *Bimin* decoction for patients with perennial allergic rhinitis: an open-label non-inferiority randomized controlled trial

**DOI:** 10.1186/s13063-019-3763-z

**Published:** 2019-12-30

**Authors:** Jingyi Zhao, Xinyu Yan, Jianqing Gai, Jinshuai Han, Hong Zhang, Hui Luo, Shaoting Huang, Junge Wang

**Affiliations:** Department of Otolaryngology, Beijing Hospital of Traditional Chinese Medicine, Capital Medical University, No. 23 Back Street of Art Museum, Beijing, 100010 People’s Republic of China

**Keywords:** Perennial allergic rhinitis, Traditional Chinese medicine, Randomized controlled trial, Clinical efficacy

## Abstract

**Background:**

Allergic rhinitis (AR) is a common allergic disease. It affects people worldwide and traditional Chinese medicine is becoming popular among AR patients because it has a definite clinical effect and there are few adverse reactions. Lung *qi* deficiency and cold syndrome (LQDCS) is a frequent type of AR, and the Chinese herbal medicine *bimin* decoction (BMD) is prescribed for it. This study compared the clinical efficacy of BMD for AR patients with LQDCS to the conventional medicine loratadine and fluticasone nasal spray.

**Methods:**

The study was an open-label non-inferiority randomized controlled trial. A total of 108 AR patients with LQDCS aged 19 to 60 were randomly allocated in a 1:1 ratio to the BMD group or the control group by the central computer system in Beijing Hospital of Traditional Chinese Medicine from January 2017 to April 2018. In total, 98 participants completed the study (BMD group *n* = 51 and control group *n* = 47). Patients in the BMD group received BMD while those in the control group received fluticasone nasal spray and loratadine tablets for 4 weeks. The primary outcome was the change in the Total Nasal Symptom Score (TNSS) between the baseline and the end of treatment. Changes in the Rhinoconjunctivitis Quality of Life Questionnaire (RQLQ), nasal resistance, and acoustic rhinometry parameters were secondary outcomes. All side effects due to the treatments were recorded.

**Results:**

After the 4-week treatment, the total TNSS was significantly reduced in both groups compared to the baseline (*P* <  0.05). No significant between-groups differences were observed for changes in TNSS scores [− 0.298 (95% confidence interval −0.640 to 0.140)], which was within the defined non-inferiority margin. RQLQ in both groups decreased significantly (*P* <  0.001) from baseline, though a more obvious reduction was observed for the BMD group (*P* <  0.001). There were no significant differences in nasal resistance, nasal volume, or nasal minimum cross-sectional area between groups after treatment (*P* > 0.05).

**Conclusions:**

These findings indicate that BMD helps relieve the symptoms of perennial AR and improves rhinitis-related quality of life. Our study indicates that BMD is non-inferior to loratadine tablets and fluticasone nasal spray for AR patients with LQDCS.

**Trial registration:**

Chinese Clinical Trial Registry, ChiCTR-INR-16010063. Registered on 2 December 2016.

## Background

Allergic rhinitis (AR) is a common allergic disease, which can be perennial or intermittent [1]. House dust mite is the most important domestic source of AR. Therapy for AR mainly includes avoiding allergens, pharmacotherapy, immunotherapy, and patient education; however, each of these has limitations [2]. The most effective treatment for AR is to avoid exposure to allergens, but airborne allergens are often difficult to avoid. Immunotherapy is not popular among patients due to its long therapy cycle (recommended for 3–5 years) and unsatisfactory results [3]. Therefore, pharmacotherapy is still the main treatment approach for AR. Although antihistamines and intranasal corticosteroids are rapid and accurate in alleviating symptoms, they do not fully regulate a patient’s immune status and sometimes have unfavorable side effects [4]. Increasingly patients are turning to complementary and alternative medicines, and thus, traditional Chinese medicine (TCM) has increased in popularity [5].

A syndrome in TCM covers a series of symptoms. The lung *qi* deficiency and cold syndrome (LQDCS) is frequently diagnosed for those with perennial allergic rhinitis (PAR). Our department has been treating AR with TCM for several decades and we have empirically formulated the herbal formula *bimin* decoction (BMD), which is composed of *Saposhnikovia divaricata* (*fangfeng*), *Astragalus* (*huangqi*), *Atractylodes* (*baizhu*), cassia twig (*guizhi*), radix paeoniae alba (*baishao*), *Prunus mume* (*wumei*), fructus chebulae (*hezi*), *Asarum heterotropoides* (*xixin*), *Schisandra chinensis* (*wuweizi*), herba ephedrae (*mahuang*), and licorice (*gancao*). BMD contains substances that have been demonstrated to have anti-inflammatory and immune regulation functions [6–9], and it is prescribed for AR patients with LQDCS. The current study aimed to compare the clinical efficacy of BMD for PAR patients with LQDCS to the clinical efficacy of the conventional medicines loratadine and fluticasone nasal spray.

## Methods

### Study design

An open-label non-inferiority randomized controlled trial was carried out to investigate the efficacy of BMD on AR symptoms, quality of life, and nasal resistance (NR) in PAR patients. All participants were recruited from the Otorhinolaryngology Department of Beijing Hospital of Traditional Chinese Medicine (BJHTCM), which is affiliated to Capital Medical University. The study design and protocol were approved by the ethics committee of BJHTCM (code 2016BL-047). The study was conducted in accordance with the principles of the Declaration of Helsinki (2004) and the Medical Research Involving Human Subjects Act. The trial was registered with the China Clinical Trial Registry Center (ChiCTR-INR-16010063) on 2 December 2016. This research will be reported according to the Consolidated Standards of Reporting Trials (CONSORT) 2010 guidelines [10].

### Participants

Recruitment information was posted in BJHTCM and publicized through the Internet. Participants with PAR who volunteered for the study were selected from January 2017 to April 2018 by physicians at otorhinolaryngology clinics. All participants had to meet the Western medicine diagnostic criteria for AR and the TCM syndrome diagnostic criteria for LQDCS. Syndrome differentiation was separately determined by two independent and qualified TCM otolaryngologists.

#### Inclusion criteria

Patients had to satisfy all of the following criteria to be included in the trial:

1. Aged 18 to 65 years, male or female
2. Experiencing the symptoms of AR (sneezing, rhinorrhea, itchy nose, and nasal obstruction) for at least 4 days per week for more than 4 weeks [11], and with a positive skin prick test to house dust mites (+++ or more, ALK reagent) according to the Allergic Rhinitis and its Impact on Asthma criteria (ARIA, 2008)
3. Syndrome differentiation corresponding to LQDCS, which is having a light pink tongue with a thin white coating and a weak pulse [12]
4. Have signed the informed consent form and volunteered to participate in the study

#### Exclusion criteria

The following were excluded from participating in the trial:

1. Women who are pregnant or hoping to conceive in the next 6 months,
2. Women who are lactating
3. Those who have nasal polyps, rhinosinusitis, an obvious deviated nasal septum, or upper respiratory tract infection
4. Those who were undergoing treatment for AR
5. Those who have serious disorders such as vascular malformation, hypertension, hematologic diseases, diabetes mellitus, malignant tumor, or mental disorders
6. Those who are allergic to the Chinese herbal medicine used

### Randomization and blinding

**Fig. 1:**
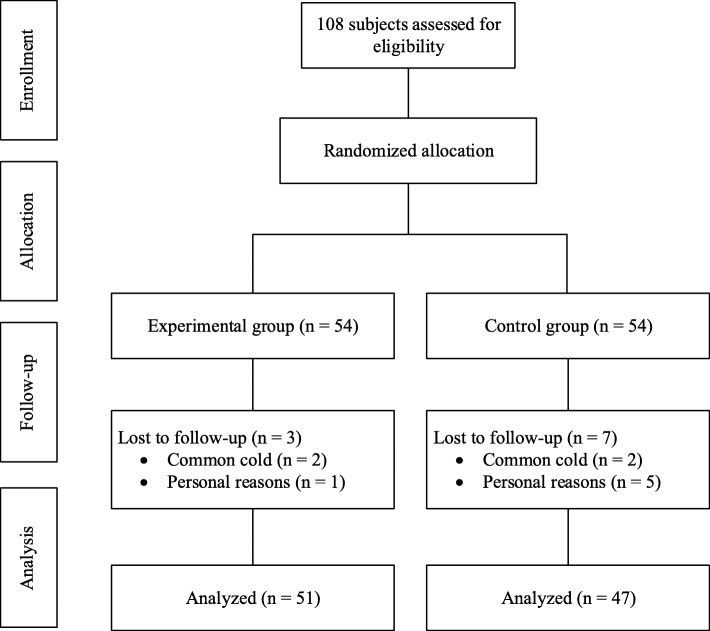
Study flow chart

The physicians were responsible for recruitment and the therapeutic assessment of patients. Participants recruited were randomly allocated into either the BMD group (*n* = 54) or the control group (*n* = 54) in a 1:1 ratio by a computer-generated random sequence in the Good Clinical Practice Office of BJHTCM after a 7-day washout period. The physicians did not have access to the sequence. The investigators were responsible for distributing the drugs. All research team members were instructed not to communicate with the participants regarding their allocation. The flow chart is in Fig. 1 and schedule is in Table 1.

**Table 1:** Study design schedule

Week	-1	0	1	2	3	4
	Baseline	Treatment and follow-up phase				
Patient enrollment	X					
Medical history	X					
Skin prick tests		X				
Informed consent		X				
Randomization		X				
TNSS		X	X			X
RQLQ		X	X			X
Adverse event recording		X	X	X	X	X

*TNSS* Total Nasal Symptoms Score, *RQLQ* Rhinoconjunctivitis Quality of Life Questionnaire

### Intervention

All participants completed the Total Nasal Symptom Score (TNSS) questionnaire and the Rhinoconjunctivitis Quality of Life Questionnaire (RQLQ), and were assessed for NR and acoustic rhinometry under instruction. The participants in the BMD group received BMD while those in the control group received fluticasone furoate spray (Flixonase, 50 μg × 120 presses, Glaxo Wellcome, S.A.) and loratadine tablets (Clarityne, Shanghai Schering Plough Pharmaceutical Co. Ltd.).

The ingredients in BMD are listed in Table 2. The herbal medicines used in the study were all produced by Beijing Institute of Traditional Chinese Medicine as a single batch. Each dose was decocted twice. All the herbal materials were soaked in cold water for 1 h before decoction. The first decoction was brought to the boil over a high heat and then simmered at a low heat for 30 min. The liquid was then filtered off. Cold water was added to the herbal materials as the second decoction, which was brought to the boil over high heat and then simmered at a low heat for 15 min. The liquid was filtered off and combined with the liquid from the first decoction to give a total volume of approximately up to 400 ml.

**Table 2:** The ingredients, dosage, and actions of herbal medicines in *bimin* decoction (enough for seven doses)

Ingredient	Dosage (g)	Actions
*Saposhnikovia divaricata* root (*fangfeng)*	10	Dispels wind-cold to prevent muscular interstices from invasion by exogenous pathogenic factors
*Astragalus* root (*huangqi)*	15	Strengthens physiological defenses and reduces edema
*Atractylodes* root (*baizhu*)	10	Consolidate the exterior of body and enhances immunologic function
Cassia twig (*guizhi*)	6	Warms *yang* and dispels cold
Radix paeoniae alba root (*baishao*)	10	Astringes acid to nourish the *yin* of the body
*Prunus mume* fruit (*wumei*)	6	Astringes lung *qi* to consolidate the base of life
Fructus chebulae fruit (*hezi*)	6	Astringes lung *qi* to consolidate the base of life
*Asarum heterotropoides* root (*xixin*)	3	Eliminates wind to disperse cold and reduce edema
*Schisandra chinensis* fruit (*wuweizi*)	6	Astringing lung *qi* to consolidate the base of life
Herba ephedrae stem (*mahuang*)	3	Relieving exterior and eliminating wind to dispersing cold
Licorice root (*gancao*)	6	Reconciling all the other herbals

Participants in the BMD group took 200 ml of BMD orally 30 min after breakfast and dinner for 4 weeks. According to the step-up therapy recommended by ARIA [13], the participants in the control group sprayed two presses per nostril of fluticasone furoate and took a 10 mg tablet of loratadine each night. No other medicines or spicy, fishy, or cold food were allowed during treatment for all participants.

Details were recorded of participants who withdrew or were excluded from the study, and their allocated medication was immediately returned to the investigators. Participants who completed the study were followed up by the physicians and at the end of the treatment period, again completed the TNSS questionnaire and RQLQ and were assessed for NR and acoustic rhinometry.

### Main outcome

The main outcome of this study was the change in TNSS. The measurement was based on four nasal symptoms (sneezing, rhinorrhea, itchy nose, and nasal obstruction). Each was scored from 0 to 3 (0 = none, 1 = mild, 2 = moderate, and 3 = severe).

### Secondary outcomes

Qualify of life was assessed with the authorized and Sinicized RQLQ, which has 28 questions on a 7-point scale (0 = not impaired at all to 6 = severely impaired) in 7 domains: (1) activity limitations, (2) sleep problems, (3) nasal symptoms, (4) eye symptoms, (5) non-nose/eye symptoms, (6) practical problems, and (7) emotional functioning [14]. The total score and seven domain scores between groups were compared.

All participants rested for 20–30 min and were required to clean up their nasal secretions before being assessed for NR and acoustic rhinometry (model: NR-6, British GM). Bilateral exhalation and inhalation resistance, total NR, and nasal minimum cross-sectional area (NMCA) were recorded. The nasal volume was calculated according to the segment 0-7cm from the anterior nostril. Each patient underwent four measurements on each side and the average was calculated for data analysis.

### Sample size

The sample size was evaluated with software SAS 9.3 (SAS Institute Inc., Cary, NC, USA) in the Clinical Evaluation Center of BJHTCM. The mean change in TNSS pre- and posttreatment was set as the indicator in the calculation. From our previous studies, we expected that the mean TNSS change for the BMD group would be 6.62 ± 2.84 and for the control group 5.79 ± 2.18 [15]. For a power of 80%, alpha of 0.05, an acceptable delta of 0.2, and a non-inferiority margin of 0.77 [15], then a clinically important difference can be detected by a sample size of at least 49 in each group. This number was then increased to 54 in each group (total of 108) to allow for a predicted 10% dropout rate.

### Statistical analysis

All statistical analyses were performed using software SPSS (SPSS Inc., Chicago, IL, USA; version 22.0) by qualified statisticians according to the intention-to-treat principle. Descriptive statistics were used to compare baseline measures and patient characteristics between groups. Least-squares mean changes from baseline were evaluated using analysis of covariance models for the primary outcome. The two-sample independent *t* test was used to compare differences in the secondary outcomes. Categorial data were assessed using Fisher’s exact test. *α* = 0.05 was defined as statistically significant.

## Results

A total of 108 patients met the criteria and were randomized into the study. Four people were eliminated because they had the common cold and six participants dropped out for personal reasons (Fig. 1). There were 51 participants (29 male and 22 female) aged 19 to 60 years (mean 36.8 years, standard deviation 11.6) in the BMD group, and 47 participants (26 male and 21 female) aged 22 to 59 years (mean 37.9 years, standard deviation 10.2) in the control group. There were no significant differences in the demographic characteristics of the groups (Table 3).

**Table 3:** Homogeneity test for general characteristics and measurement variables at baseline (mean ± standard deviation)

Characteristic	*Bimin* decoction group (*n* = 51)	Control group (*n* = 47)	푃 value
Age (years)	36.8 ± 11.6	37.9 ± 10.2	0.523
Male/female	29/22	26/21	0.221
TNSS (score)			
Overall	7.84 ± 1.46	8.43 ± 1.56	0.062
Sneezing	2.22 ± 0.67	2.28 ± 0.71	0.608
Runny nose	2.06 ± 0.79	2.23 ± 0.76	0.265
Itchy nose	1.90 ± 0.94	2.19 ± 0.74	0.141
Nasal obstruction	1.67 ± 0.95	1.72 ± 0.85	0.784
RQLQ (score)			
Overall	67.18 ± 8.19	66.81 ± 9.23	0.735
Activity limitations	9.04 ± 2.88	9.77 ± 3.10	0.226
Sleep problems	6.12 ± 2.42	6.36 ± 2.34	0.576
Nasal symptoms	14.24 ± 2.95	13.94 ± 3.00	0.604
Eye symptoms	5.80 ± 3.02	4.85 ± 3.20	0.128
Non nose/eye symptoms	14.04 ± 3.48	13.72 ± 3.75	0.702
Practical problems	10.25 ± 2.54	11.04 ± 3.00	0.123
Emotional functioning	7.69 ± 2.67	7.13 ± 2.94	0.331

*TNSS* Total Nasal Symptoms Score, *RQLQ* Rhinoconjunctivitis Quality of Life Questionnaire

### TNSS

**Fig. 2:**
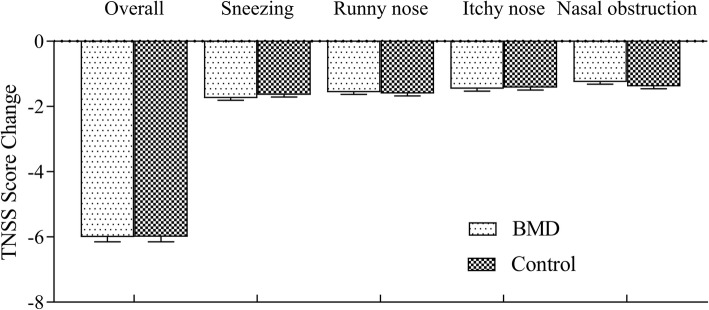
Mean of total nasal symptom score for the 4-week treatment period. Datas were compared with baseline. TNSS, Total Nasal Symptoms Score. BMD, *Bimin* decoction

The pretreatment TNSS scores were similar in both groups (BMD group 7.84 ± 1.46, control group 8.43 ± 1.56; *P* = 0.062). After the 4-week treatment, the total TNSS score fell for both groups: for the BMD group from 7.84 ± 1.46 to 2.17 ± 1.26 (*P* = 0.019) and for the control group from 8.43 ± 1.56 to 2.29 ± 0.93 (*P* = 0.021) (Tables 3 and 4). The 95% confidence interval for group mean change difference was − 0.640 to 0.140, which is within the defined non-inferiority margin of 0.77 (Table 4 and Fig. 2).

**Table 4:** Effect of treatments on allergic rhinitis symptoms

TNSS (score)	Least squares mean change from baseline (± standard error)		Mean difference (95% confidence interval)	푃 value
	*Bimin* decoction group (*n* = 51)	Control group (*n* = 47)		
Overall	6.002 ± 0.149	5.997 ± 0.155	−0.298 (−0.640 to 0.104)	0.982
Sneezing	1.742 ± 0.068	1.642 ± 0.071	0.066 (− 0.182 to 0.314)	0.316
Runny nose	1.560 ± 0.072	1.605 ± 0.075	−0.150 (− 0.425 to 0.126)	0.666
Itchy nose	1.454 ± 0.074	1.422 ± 0.077	− 0.179 (− 0.505 to 0.147)	0.763
Nasal obstruction	1.252 ± 0.067	1.386 ± 0.070	−0.169 (− 0.466 to 0.128)	0.171

*TNSS* Total Nasal Symptoms Score

### RQLQ

After the treatment, the single and overall RQLQ scores fell significantly for the two groups: for the BMD group from 67.18 ± 8.19 to 14.54 ± 3.56 (*P* <  0.001) and for the control group from 66.81 ± 9.23 to 22.45 ± 4.70 (*P* < 0.001). The fall in RQLQ total score for both groups after treatment was statistically significant (*P* < 0.001) (Table 5).

**Table 5:** Effect of treatments on Rhinoconjunctivitis Quality of Life Questionnaire scores (mean ± standard deviation)

RQLQ (score)	*Bimin* decoction group (*n* = 51)	Control group (*n* = 47)	*P* value
Overall	14.54 ± 3.56	22.45 ± 4.70	<0.001
Activity limitation	2.92 ± 1.56	2.81 ± 1.79	0.170
Sleep problems	0.90 ± 1.01	1.85 ± 1.16	<0.001
Nasal symptoms	2.75 ± 1.75	4.26 ± 1.87	<0.001
Eye symptoms	1.20 ± 1.39	1.55 ± 1.64	0.319
Non nose/eye symptoms	3.71 ± 2.54	6.19 ± 2.74	<0.001
Practical problems	2.02 ± 1.49	3.79 ± 2.27	<0.001
Emotional functioning	1.08 ± 1.07	2.00 ± 1.63	0.004

*RQLQ* Rhinoconjunctivitis Quality of Life Questionnaire

### Nasal resistance and acoustic rhinometry

There were no significant differences in the change in NR, nasal volume, or NMCA between groups (*P* > 0.05) (Tables 6 and 7).

**Table 6:** Comparison of nasal resistance between groups after treatment (Pa/cm^3^, mean ± standard deviation)

	*n*	Inhalation resistance						Exhalation resistance					
		T1			T2			T1			T2		
		Left	Right	Total	Left	Right	Total	Left	Right	Total	Left	Right	Total
Experimental group	51	1.55 ± 0.56	1.48 ± 0.58	0.73 ± 0.24	0.73 ± 0.26	0.66 ± 0.26	0.36 ± 0.12	1.47 ± 0.58	1.79 ± 0.61	0.71 ± 0.24	0.69 ± 0.23	0.66 ± 0.21	0.32 ± 0.10
Control group	47	1.63 ± 0.63	1.61 ± 0.62	0.78 ± 0.29	0.77 ± 0.28	0.76 ± 0.26	0.37 ± 0.12	1.48 ± 0.57	1.55 ± 0.59	0.73 ± 0.25	0.70 ± 0.24	0.68 ± 0.22	0.33 ± 0.11
*P*		0.27	0.393	0.126	0.685	0.521	0.821	0.621	0.932	0.477	0.569	0.606	0.526

*T1* baseline at subject recruitment, *T2* 1 day after intervention

**Table 7:** Comparison of acoustic rhinometry between groups after treatment (mean ± standard deviation)

	*n*	Nasal volume (ml)				Nasal minimum cross-sectional area (cm^2^)			
		T1		T2		T1		T2	
		Left	Right	Left	Right	Left	Right	Left	Right
Experimental group	51	5.32 ± 1.18	5.34 ± 1.11	8.04 ± 1.83	8.25 ± 1.80	0.39 ± 0.19	0.34 ± 0.15	0.63 ± 0.19	0.56 ± 0.16
Control group	47	5.25 ± 1.19	5.35 ± 1.20	8.09 ± 1.92	8.31 ± 2.05	0.32 ± 0.15	0.38 ± 0.11	0.62 ± 0.21	0.55 ± 0.18
*P*		0.941	0.163	0.92	0.38	0.655	0.163	0.741	0.316

*T1* baseline at subject recruitment, *T2* 1 day after intervention

### Safety

Both treatments were well tolerated. Seven patients reported a total of 13 adverse events (BMD group 5, control group 8): dry nose (2), sore throat (2), or sleepiness (1) for the BMD group, and dry nose (4), sore throat (3), or coughing (1) for the control group. None of the adverse events were serious and all were resolved with or without treatment.

## Discussion

Modern drug treatments for PAR mainly include antihistamines and intranasal corticosteroids [2]. These medicines have different roles in the prevention and therapy of AR. Although these medications have clear targets, act rapidly, and have pronounced effects, they have different disadvantages to various degrees. For example, intranasal corticosteroids must be used continuously for several days to achieve the maximum effect and then gradually reduced to the minimum dose to control symptoms. However, patients may discontinue treatment when their symptoms appear to be relieved. Antihistamines also have side effects, such as cardiac toxicity, drowsiness, and operational disability [16]. As a result, more and more clinicians and patients are looking for complementary alternative medicines, such as Chinese herbal medicines, to treat AR. TCM treatments not only control the clinical symptoms but also regulate the constitution.

AR is a significant disease in TCM, coming under the classification *biqiu* (鼻鼽). According to TCM, AR is caused by a specific constitutional state, depletion of viscera, and exogenous pathogenic factors. Once the inducing factors have been diagnosed, the symptoms are easy to treat. There is a long history of using TCM to treat AR. TCM can regulate the immune function to relieve symptoms and reduce the frequency of attacks. Research has shown that an ethanolic extract of *Asarum heterotropoides* (*xixin*) reduces anaphylaxis, and has anti-allergic effects like those of antihistamines [17]. *Schisandra chinensis* (*wuweizi*) increases the production of lymphoblastic cells and enhances the immune function. It promotes DNA synthesis by lymphocytes through its lung astringe and kidney nourishment function [18, 19]. Licorice root (*gancao*) contains glucocorticoids that likely have anti-inflammatory and anti-allergic effects. Its main components are flavonoids and licorice compounds, which may alleviate the cardiac toxicity and side effects of antihistamines [19, 20].

This study evaluated the efficacy of BMD and indicated that it is non-inferior to antihistamines and intranasal corticosteroids. The differences in changes of TNSS score in the BMD group and the control group after treatment were not statistically significant. The fall in the total RQLQ score after treatment in the BMD group was significantly lower than that of the control group. The obvious improvements relating to sleep, work, and overall comfort indicate the advantages of BMD in improving systemic symptoms. No significant differences in changes of total NR, nasal cavity volume, or NMCA were observed in either group after treatment. We found that BMD alleviates mucosal hyperemia, edema, and nasal turbinate swelling, leading to a reduction of NR and an increase of NMCA. Subsequent research will examine any change in the number of lymphocytes in AR patients after BMD therapy.

## Conclusions

This study indicates that BMD is non-inferior to a combination of a fluticasone nasal spray and loratadine in alleviating AR symptoms. Quality of life in the BMD group relating to sleep, work, and overall comfort was significantly better than in the control group. BMD may be a good alternative medicine for AR patients considering its satisfactory efficacy and better quality of life.

## Data Availability

The supporting data are available from the corresponding author on reasonable request.

## Abbreviations

AR: Allergic rhinitis
ARIA: Allergic Rhinitis and its Impact on Asthma project
BJHTCM: Beijing Hospital of Traditional Chinese Medicine
BMD: *Bimin* decoction
LQDCS: Lung *qi* deficiency and cold syndrome
NMCA: Nasal minimum cross-sectional area
NR: Nasal resistance
PAR: Perennial allergic rhinitis
RQLQ: Rhinoconjunctivitis Quality of Life Questionnaire
TCM: Traditional Chinese medicine
TNSS: Total nasal symptom score
